# Feasibility of dairy waste water (DWW) and distillery spent wash (DSW) effluents in increasing the yield potential of *Pleurotusflabellatus* (PF 1832) and *Pleurotus sajor*-*caju* (PS 1610) on bagasse

**DOI:** 10.1007/s13205-012-0053-9

**Published:** 2012-03-21

**Authors:** Ritu Gothwal, Aditi Gupta, Ashwani Kumar, Satyawati Sharma, B. J. Alappat

**Affiliations:** 1 Department of Civil Engineering, Indian Institute of Technology, Hauz Khas, Delhi, 110016 India; 2 Centre for Rural Development and Technology, Indian Institute of Technology, Hauz Khas, Delhi, 110016 India

**Keywords:** Bagasse, *Pleurotus* spp., Dairy waste water (DWW), Distillery spent wash (DSW)

## Abstract

In the present investigation, feasibility of dairy waste water (DWW) and distillery spent wash (DSW) effluents in increasing the growth and yield of two species of oyster mushroom, *Pleurotus flabellatus* (PF 1832) and *P. sajor*-*caju* (PS 1610) on abundantly available agro-waste, bagasse, was evaluated. Three different levels of treatments were applied for each effluent. The effects of amendments on the result were observed in terms of yield, biological efficiency (BE) and substrate dry-matter loss. BE was found to be the highest (66.63 ± 1.0 %) for *P. sajor*-*caju* grown on bagasse amended with 10 % DWW and lowest for *Pleurotus* controls. While *P. sajor*-*caju* performed better on bagasse amended with DWW, *P. flabellatus* was more suited to grow on DSW amended bagasse. Degradation of complex molecules was in accordance with substrate dry-matter loss and the respective yields. The biochemical analysis of mushroom fruit bodies showed them to be a rich source of protein (maximum 36.40 %) and sugars (maximum 41.58 %). The study thus proved to be beneficial for effective management of the waste by employing higher order fungi as well as obtaining nutrient-rich delicacy for the mass.

## Introduction

Bioconversion of agro-industrial wastes into value-added products is gaining considerable importance in the recent years (Dashtban et al. 2009; Ingale and Ramteke 2010; Philippoussis and Diamantopoulou 2011). Concerted efforts are being made worldwide to switch their status from ‘waste’ to ‘new resources’ (Philippoussis and Diamantopoulou 2011).

Mushroom cultivation offers a highly efficient method capable of not only biodegradation and bioremediation of agro-industrial waste but also biotransformation into proteinaceous food that can sustain food security in the developing countries (Bisaria et al. 1987; Ingale and Ramteke 2010; Kuforiji and Fasidi 2009; Kulshreshtha et al*.*^2010^; Mane et al. 2007; Narain et al. 2011; Philippoussis and Diamantopoulou 2011). A much smaller group of the filamentous fungi, oyster mushrooms of genus *Pleurotus,* are efficient colonizers and bioconverters of lignocellulosic agro-industrial residues into palatable human food with organoleptic properties and nutritive value (Loss et al. 2009; Philippoussis et al. 2001). Due to a large variety of non-specific lignocellulosic enzymes produced by the *Pleurotus* spp., they can be cultivated on a number of agricultural wastes. Although paddy straw and wheat straw are the traditional substrates for *Pleurotus* spp., different biological efficiencies have been reported by various authors, viz., 11.66, 35.42–46.60 and 128 % for the former (Bisaria et al. 1987; Ragunathan et al. 1996; Zhang et al. 2002) and 11.07, 75–100 and 97 % for the latter (Bisaria et al. 1987; Kirbag and Akyuz 2008; Philippoussis and Diamantopoulou 2011; Zhang et al. 2002). Other non-traditional substrates used alone or in combination with the traditional substrates include cotton stalks (BE, 32.69–41.42 %) maize stover (BE, 25.18–35.39 %), coir pith (BE, 26.11–27.33 %), sorghum stover (BE, 32.17–36.84 %), saw dust (BE, 73.5 %), banana leaves (BE, 10.25 %), mango leaves (BE, 5.96 %), bagasse (BE, 34.29–41.31 %), peanut shell, corn cobs, coffee pulp, *Populus deltoides*, *Eupatorium adenophorum*, etc. (Bisaria et al. 1987; Ingale and Ramteke 2010; Madan et al. 1987; Moonmoon et al. 2010; Patrabansh and Madan 1997; Ragunathan and Swaminathan 2003; Sharma et al*.*^2001^). Among these residues is sugarcane bagasse, one of the major by-products of sugarcane industries generated in abundance in countries like India, Brazil, Egypt, etc. Several authors have utilized bagasse for the production of *Pleurotus* mushrooms and found that it could be used as a viable substrate for the mass production of the same, either alone or with the addition of certain supplements (Bisaria et al. 1987; El-Sayed et al. 1994; Moda et al. 2005).

Substrate supplementation is a practice that shortens the crop period and also increases mushroom productivity (Madan et al. 1987; Narain et al. 2011). Recently, few studies have considered the exploitation of agro-industrial effluents as additives for the growth of *Pleurotus* spp. mushrooms (Kalmis and Sargin 2004; Loss et al. 2009; Narain et al. 2011; Pant et al. 2006; Philippoussis 2009; Ruiz-Rodriguez et al. 2010). The fact that these effluents are a rich source of organic matter and nutrients (carbon, nitrogen, phosphorous, potassium, etc.) makes them quite useful as substrate supplements that may help in the augmentation of mushroom yield. For example, Narain et al. (2011) showed that low concentrations of dairy waste water (DWW), which is rich in suspended solids, proteins, milk fat and other organics, could increase the fruit body yield and biological efficiency (max 108.68 % at 10–20 % supplementation) of two *Pleurotus* spp. grown on wheat straw and corn cobs as basal substrates. Similarly, distillery spent wash (DSW), besides showing beneficial effects on plants/cereals, has also been utilized as a substrate amendment in the practice of mushroom cultivation. Pant et al. (2006) have suggested the use of wheat straw amended with spent wash effluent to increase the yield of the *P. florida* and *P. pulmonarius* (max BE 238.6 % for *P. florida*). These industrial effluents thus find a cheaper but an efficient way of disposal.

The present study aims to investigate the influence of DWW and DSW effluents as additives on the growth and yield of *P. flabellatus* and *P. sajor*-*caju* on bagasse. It also aims to identify the appropriate strains that are well suited to grow on DWW and DSW amended bagasse substrate. The study would thus be useful for bioremediation of the above-mentioned residues along with production of proteinaceous food for the mass.

## Materials and methods

### Preparation of substrate

The experiment was conducted over a span of 2 months from middle of November to the middle of January, when there was no availability of bagasse in the Indian sugar mills. Therefore, bagasse was collected from a local juice shop at Jaipur, India. It was chopped into 3–4 cm pieces and dried in the oven at 60 °C. DWW was obtained from Saras Dairy at Jaipur, Rajasthan and DSW from Dhampur sugar mill industry, Delhi, India. These effluents were stored in glass containers at −20 °C for the subsequent use. Prior to their use, these effluents were autoclaved at 121 °C and 15 psi for 20 min to make them free from any source of contamination.

### Mushroom spawn

The grain spawn of *P. flabellatus* (PF 1832) and *P.sajor*-*caju* (PS 1610) were procured from Bharat Mushroom Organisation, New Delhi, India.

### Experimental setup

Completely dried, chopped bagasse substrate (4–5 cm) was soaked overnight with water containing formalin (100 ml formalin in 100 l water). After this, the bagasse was taken out, drained off excess water and then evenly spread on a formalin-cleaned plastic sheet. It was allowed to dry to obtain an average moisture content of 60 ± 1 %, calculated by drying 100 g of this bagasse in an oven at 70 °C until constant weight was achieved.

2.5 kg wet bagasse (equivalent to 1 kg dry bagasse; moisture content 60 %) was taken and the required amounts of each amendment (DWW and DSW) on dry-weight basis were added and mixed properly. Table 1 lists the various substrate combinations designed for each *Pleurotus* spp. These amended substrates along with subsequent layers of mushroom grain spawn (10 % of dry weight) and 5 ml *Azotobacter* broth (pH = 7; cfu ~ 10^8^ cells/ml), taken in place of gram powder as a source of nitrogen (Eyini et al. 2005), were filled in the polythene bags (28 cm × 20 cm). Holes were made in these polythene bags for proper aeration. Table 1 gives properties of the substrate combinations before inoculation with *Pleurotus* spp. Substrate combinations without the amendments, but containing *Azotobacter* broth and each of the *Pleurotus* spp. were taken as the respective controls.

**Table 1:** Substrate combinations designed for each *Pleurotus* spp. and their properties prior to inoculation

Serial no.	Amendment in the substrate combination (bagasse + *Azotobacter* + amendment)	Moisture content (%) in the substrate combination	Nitrogen content (%) of the substrate combination	pH of the substrate combination
1	1 % DWW	60 ± 0.5	1.30 ± 0.03	6.72 ± 0.03
2	5 % DWW	61 ± 0.3	1.32 ± 0.02	6.84 ± 0.05
3	10 % DWW	63 ± 1.2	1.34 ± 0.04	6.96 ± 0.02
4	1 % DSW	60 ± 0.7	1.31 ± 0.02	6.65 ± 0.04
5	5 % DSW	62 ± 0.5	1.33 ± 0.02	6.73 ± 0.03
6	10 % DSW	63 ± 0.8	1.35 ± 0.03	6.79 ± 0.06

Values indicated are average of three determinations

The polythene bags were kept in a sterilized room with its temperature varying between 17 and 22 °C. Spawn was run at 20–22 °C for about 3–4 weeks. After the completion of spawn run, which was visible from the transparent polythene sheet as a white mycelial growth over the substrate, the polythene bags were cut open. Water was sprinkled regularly twice, at morning and afternoon, to develop fruit bodies for about a period of 3 weeks. Mushrooms were harvested in three flushes, each separated by a time interval of 4–6 days.

### Yield and biological efficiency

The mushroom fruit bodies were harvested and fresh weight was noted. The biological efficiency was calculated using the following formula (Bisaria et al*.*^1987^):

### Chemical analysis

Pre and post chemical analysis (50 days after inoculation) of amended bagasse substrate was done. Estimation of cellulose was done by acetolysis followed by hydrolysis to form glucose units. These glucose units were then dehydrated and reacted with anthrone to give a green colour product, absorbance of which was measured at 630 nm using a spectrophotometer (Thimmaiah 1999). Hemicellulose and lignin were estimated by determining neutral detergent fibre (NDF) and acid detergent fibre (ADF) (Thimmaiah 1999).

For the effluents, pH and EC were measured using pH meter (Eutech Instruments pH 510) and EC meter, respectively (Eutech Instruments CON 510), and total solids and volatile solids were calculated by heating the samples in the oven (60–70 °C) and muffle furnace (550 ± 5 °C), respectively. Chemical oxygen demand (COD) was calculated following the protocol given by Pitwell (1983).

Nitrogen content of bagasse, effluents and mushroom fruit bodies was estimated by the CHN analyzer (CHNOS Elementar, Vario EL III model). Flame photometer and spectrophotometer were used for the determination of available potassium (K) and available phosphorus (P), respectively (Rowell 1994). Moisture content of the mushroom fruit bodies was calculated by heating them in the oven at 60–70 °C. After drying the mushroom fruit bodies completely, ash content was obtained by heating them at 550 ± 5 °C for about 5 h in a muffle furnace. The crude protein was calculated by multiplying the nitrogen content by a factor of 4.38 (Fitzpatrick et al. 1946). The total soluble sugars in the fruit bodies were estimated using the Anthrone method (Thimmaiah 1999). Fat content was measured using hexane as the extracting solvent and heating at 60–65 °C in a soxhlet apparatus.

### Statistical analysis

The data, collected in triplicates, was analyzed by one way analysis of variance (ANOVA) using SPSS for windows (version 18.0). The significance of difference was determined according to Duncan’s multiple range test (DMRT). *P* values <0.05 were considered to be statistically significant.

## Results and discussion

### Pre analysis of the substrate

Chemical composition of the bagasse has been shown in Table 2. The various physico-chemical parameters of DWW and DSW have been reported in Table 3. Table 1 gives properties of the various substrate combinations prior to inoculation with *Pleurotus* species. The moisture content of all the combinations varied between 60 and 63 % and the nitrogen content between 1.30 and 1.35 %. It can be seen from Tables 2 and 3, that bagasse has a little acidic pH whereas the effluents are slightly alkaline in nature. However, on mixing the two in the proportions designed, the pH ranged between 6.65 and 6.96 for all the substrate combinations (Table 1), which was desirable for the mushroom growth (Kalmis and Sargin 2004).

**Table 2:** Composition of bagasse

S. No.	Components	Composition
1	Cellulose	51.3 ± 1.2 %
2	Hemi cellulose	33.5 ± 0.78 %
3	Lignin	8.2 ± 0.23 %
4	Total nitrogen	1.28 ± 0.04 %
5	Carbon	28.76 ± 0.03 %
6	Available phosphorous	0.27 ± 0.38 %
7	Available potassium	0.5 ± 0.003 %
8	C/N ratio	22.46
9	pH	6.6 ± 0.07

Values indicated are average of three determinations

**Table 3:** Physico–chemical parameters of effluents

Parameter	Dairy waste water (DWW)	Distillery Spent wash (DSW)
pH	7.87	7.15
Electrical conductivity (EC) (μS cm^−1^)	2,680 ± 14	27,350 ± 26
Total solids	3,847 ± 18	32,486 ± 13
Volatile solids	2,465 ± 17	27,350 ± 21
COD	3,250 ± 23	29,740 ± 54
Total nitrogen	292 ± 6	597 ± 9
Available phosphorous	38 ± 2.3	25 ± 1.5
Available potassium	61 ± 4.8	73 ± 2.3

All parameters except pH and EC expressed in mg l^−1^

Values indicated are average of three determinations

### Mushroom’s growth on amended substrate

The growth of the mushrooms on bagasse amended with DWW and DSW effluents was monitored in terms of yield, biological efficiency and substrate dry-matter loss. The effluents were tested at a concentration of 1, 5 and 10 % since at higher concentrations; there were increased risks of contamination.

#### Bagasse amended with Dairy waste water (DWW)

**Fig. 1:**
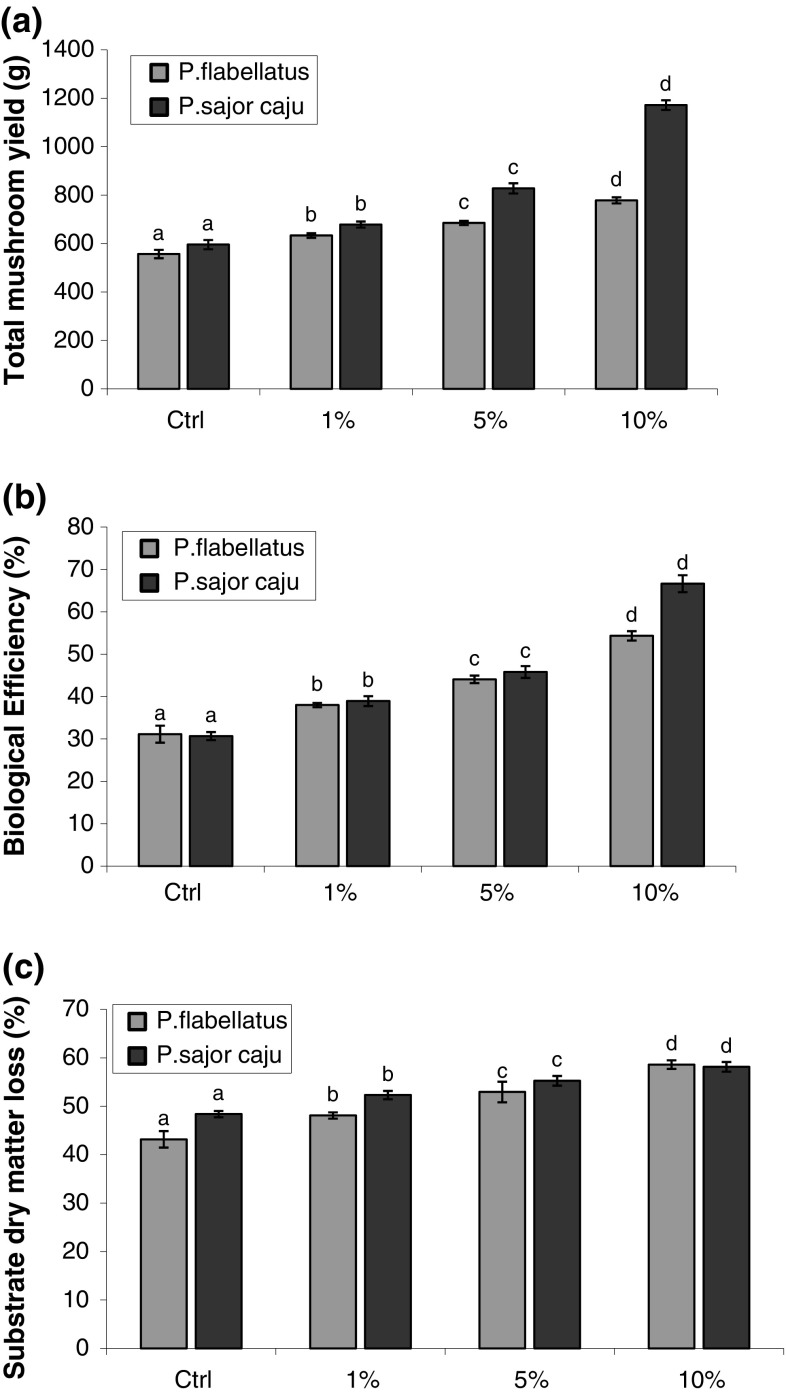
Graphs showing **a** yield, **b** biological efficiency (BE) and **c** substrate dry-matter loss of *Pleurotus* spp. grown on bagasse amended with different concentrations of dairy waste water (DWW). *Vertical bars* over the histogram indicate standard deviation (SD). *Letters* above the histogram bars indicate analysis of variance (ANOVA). *Bars with same letters* are statistically not different from each other (*P* < 0.05) using DMRT for a particular species

Significant difference was observed in yield and biological efficiency for the two mushroom species with respect to different usage of the dairy effluent concentrations (Fig. 1a, b). Overall, maximum biological efficiency (BE) of 66.63 ± 1.0 % was obtained for *P. sajor*-*caju* grown on bagasse amended with 10 % DWW. The value of the highest biological efficiency obtained in our study is more than the one obtained by Bisaria et al*.* (1987) who reported a value of 8.33 % for the growth of *P. sajor*-*caju* on bagasse and by Pant et al. (2006) where biological efficiency for the cultivation of *P. florida, P. sajor*-*caju* and *P. pulmonaris* on bagasse varied between 33.9 and 46.9 %. However, Narain et al. (2011) observed that highest BE’s of 108.68 and 91.27 % could be obtained for *P. florida* PF05 and *P. sajor*-*caju* PS08 grown on wheat straw and corn cob substrate combination (1:1) supplemented with 10 and 20 % DWW, respectively. Nevertheless, for both the mushroom species used in the present study, the biological efficiency increased with increasing concentrations of DWW (Fig. 1b). Although, bagasse is already rich in sugars and nitrogen (Table 2), supply of extra nutrients was ensured through DWW supplementation. DWW is rich in nitrogen and contains considerable amounts of lactose, milk fat, protein and lactic acid, phosphates, calcium, sulphate, potassium, other minerals and some biodegradable constituents (Narain et al. 2011).The presence of these nutrients in DWW thus accounts for its growth/yield promoting effects. This can be fully supported by the work of Narain et al. (2011) where DWW supplementation (5–20 %) increased the growth rate, ergosterol production, mycelia run, pin head formation and yield of *P. florida* PF05 and *P. sajor*-*caju* PS08. Higher concentrations (>20 %) were inhibitory due to the presence of excess nitrogen which inhibited fungal growth and fruiting (Curvetto et al. 2002; Narain et al. 2009, 2011).

Significant differences in the weight loss over the control were observed for both the *Pleurotus* spp. (Fig. 1c). Maximum substrate dry-matter loss, i.e., 58.60 ± 0.44 % was observed for *P. sajor*-*caju* grown on bagasse amended with 10 % DWW while a minimum loss of 43.17 ± 1.70 % was observed for *P*. *flabellatus* control. These values correspond well with the respective yields and biological efficiencies suggesting that the yields of the mushroom fruit bodies were proportionate to the substrate dry-matter losses. These findings also get supported by the work of Pant et al. (2006).

#### Bagasse amended with DSW

**Fig. 2:**
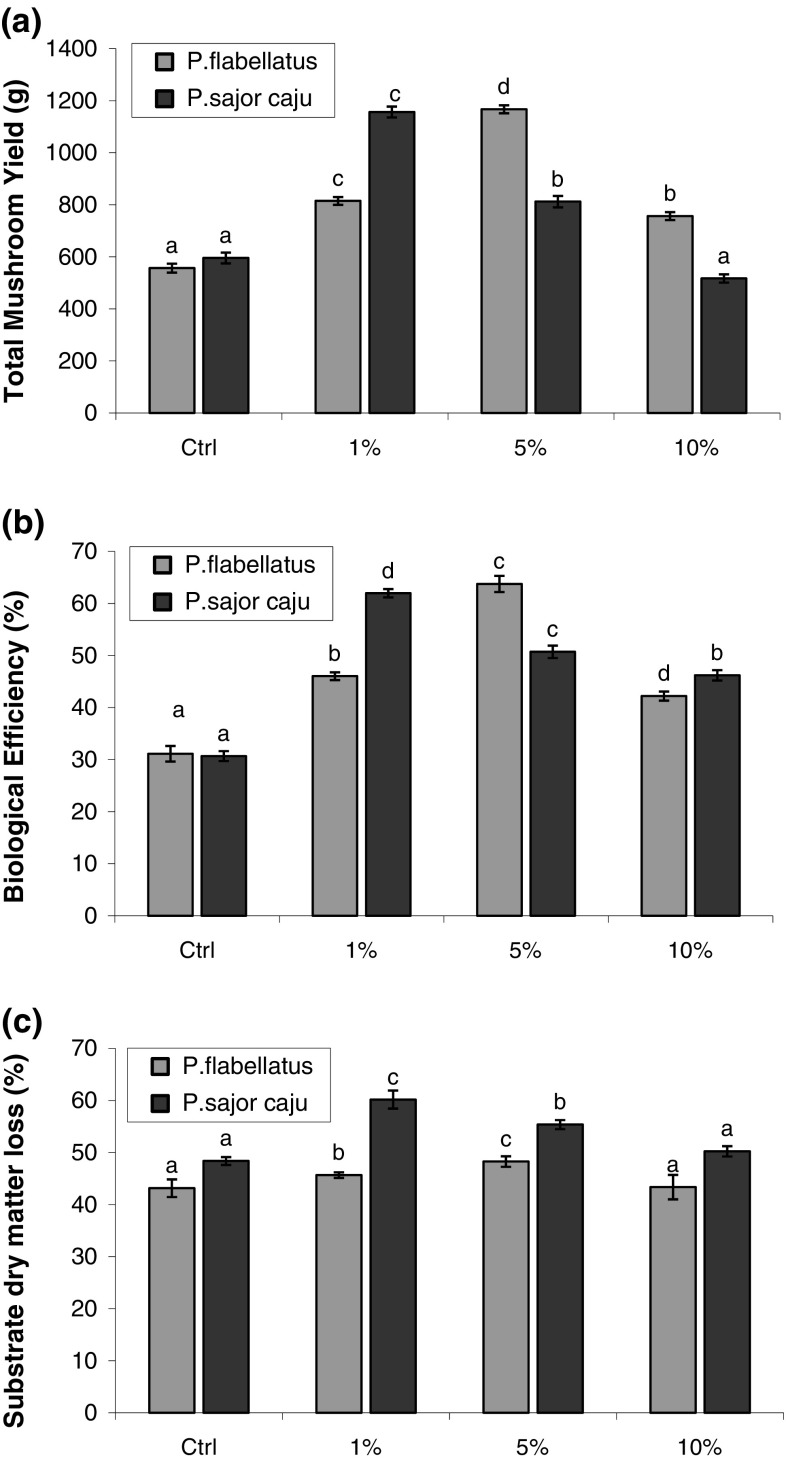
Graphs showing **a** yield, **b** biological efficiency (BE) and **c** substrate dry-matter loss of *Pleurotus* spp. grown on bagasse amended with different concentrations of distillery spent wash (DSW). *Vertical bars* over the histogram indicate standard deviation (SD). *Letters* above the histogram bars indicate analysis of variance (ANOVA). *Bars with same letters* are statistically not different from each other (*P* < 0.05) using DMRT for a particular species

The total yields of the two mushroom species cultivated on bagasse amended with different concentrations of DSW have been shown in Fig. 2a. With DSW, maximum biological efficiency of 63.75 ± 0.48 % was obtained for *P. flabellatus* grown on bagasse with 5 % DSW and a minimum for the *P. sajor*-*caju* control. This value of highest BE observed with DSW in our present study is more than that obtained by Pant et al. (2006) who reported BE between 33.9 and 46.9 % for bagasse but less than that reported for wheat straw (33.1–238.6 %), when both were amended with different concentrations of DSW for the growth of *Pleurotus* spp. studied. In case of *P. flabellatus* in the present study, BE increased significantly over the control on 1 % addition of DSW and further on 5 % addition. However, the addition of 10 % amendment caused a significant dip in BE of the same. On the other hand, in case of *P. sajor*-*caju*, BE increased sharply over the control on addition of 1 % DSW but decreased on further addition of 5 and 10 % amendment (Fig. 2b). This may be due to the fact that although spent wash was beneficial at lower concentration, it inhibited the growth of the mushrooms at higher concentrations. Similar observations were recorded by Pant et al. (2006) where productivity of *P. sajor*-*caju* and *P. pulmonarius* was enhanced at lower concentrations but declined at higher concentrations (>50 %) of DSW. Many inhibitory effects with higher concentrations of DSW have been reported in plants as well (Manunatha 2008; Rath et al. 2010). Such inhibitory effects might be attributed to high electrical conductivity (EC) of DSW (>20 mS/cm) where excess of various forms of cations and anions might be responsible for the reduction in the growth/yield (Manunatha 2008; Rath et al. 2010). Ayodele and Ojoghoro (2007) reported the effect of different salt concentration on vegetative growth performance of *P. tuberregium*, where they showed that the mushroom can tolerate or utilise a wide range of salts at different concentrations. It was observed that vegetative growth and development of *P. tuberregium* was inhibited mostly by NaCl, MgCl and KCl, whereas less inhibition was observed in MgSO_4_, Na_2_SO_4_ and K_2_SO_4_. The inhibitory effect was more with chloride salts compared with sulphate salts at concentrations of 15 and 20 %. This may be due to the interference with uptake of water by the salts which is very essential for growth and development of mushrooms.

Overall, maximum yield of mushroom fruit bodies from bagasse amended with 10 % DWW can be explained due to the fact that DWW is a rich source of nutrients and can be easily tolerated up to 10 % concentration. 10 % inclusion might have provided enough nutrients suitable for the growth of mushroom fungi. Although DSW has a better nutrient content (Rath et al. 2010; Suganya and Rajannan 2009), its increased concentration (10 %) becomes inhibitory for the growth of mushroom, and therefore, even the availability of better nutrient supply becomes unfavorable. The use of DSW at lower concentrations might have been a poor source of nutrients compared to DWW effluent which could be suitably tolerated up to 10 % inclusion. Thus, some of the macro and micro-nutrients, which are important for plant growth and yield, become unfavorable, beyond tolerance limit, and cause adverse effects (Baghel 2008).

The differences in the substrate dry-matter loss were significant between control, 1 and 5 % addition of DSW effluent but non-significant at 10 % addition for both the *Pleurotus* spp. However, the trend within the same species for substrate dry-matter loss was similar to that observed for yield and biological efficiency (Fig. 2c).The substrate dry-matter losses showed a maximum of 60.18 ± 1.73 % for *P. sajor*-*caju* grown on bagasse with 1 % DSW amendment and a minimum for the *P. flabellatus* control.

### Biochemical analysis of mushroom fruit bodies

The biochemical composition of fruit bodies (average of three flushes) tested has been reported in Table 4. The crude protein in *Pleurotus* spp. varied between 27.86 to 36.40 % with a maximum for *P. sajor*-*caju* grown on bagasse with 10 % DSW. The values fall well within the range of protein content of *P. sajor*-*caju* (26.3–36.7 %) grown on different agro-wastes as studied by Bisaria et al. (1987). The protein content of the mushroom fruit bodies varies with the kind of the substrate chosen due to the differential nature of the nutrient supply (Bisaria et al. 1987; Bonatti et al. 2004). On an average, mushrooms grown on bagasse amended with DSW were found to be more proteinaceous than grown with DWW amendment. This might be due to the fact that DSW has higher nitrogen content as compared with the DWW (Rath et al. 2010). The same has been observed in our study also (Table 3). Bagasse already contains a large amount of residual sugar and some nitrogen which is readily available as a nutrient source for the fungus (Pant et al. 2006). However, supplementation of the substrate with other nitrogen/protein rich effluents might have led to increase in yield as well as nutrient content (specifically protein content) of the mushroom fruit bodies. Our results in this regard can also be corroborated by the findings of Narain et al. (2011) where low concentrations of DWW (up to 20 %) enhanced the protein content in the fruit bodies of *P. florida* and *P. sajor*-*caju* and by Sukanya and Meli (2003) and Manunatha (2008), where low concentration of DSW as a nitrogen source improved the growth, yield and quality of the crops studied.

**Table 4:** Biochemical Analysis of mushroom fruit bodies grown on bagasse amended with DWW and DSW

	Crude protein (%)		Total soluble sugars (%)		Moisture (%)		Ash (%)		Fat (%)	
	*P. flabellatus*	*P. sajor*-*caju*	*P. flabellatus*	*P. sajor*-*caju*	*P. flabellatus*	*P. sajor*-*caju*	*P. flabellatus*	*P. sajor*-*caju*	*P. flabellatus*	*P. sajor*-*caju*
(a) DWW amendment										
Control	27.86 ± 0.36^a^	30.98 ± 0.15^a^	31.95 ± 0.35^a^	31.41 ± 0.51^a^	88.72 ± 0.78^a^	88.46 ± 0.25^a^	8.28 ± 0.24^a^	8.06 ± 0.24^a^	1.75 ± 0.05^c^	1.73 ± 0.03^c^
1 %	29.53 ± 0.98^b^	32.87 ± 0.56^b^	36.79 ± 0.36^b^	35.19 ± 0.30^b^	90.20 ± 0.21^b^	90.06 ± 0.15^b^	8.19 ± 0.78^a^	8.15 ± 0.85^a^	1.73 ± 0.07^b^	1.68 ± 0.06^a^
5 %	31.04 ± 0.25^c^	33.62 ± 0.70^c^	38.33 ± 0.78^c^	38.16 ± 0.55^c^	91.85 ± 0.45^c^	88.93 ± 0.30^a^	8.23 ± 1.78^a^	8.18 ± 1.77^a^	1.71 ± 0.02^a^	1.70 ± 0.03^b^
10 %	33.40 ± 1.80^d^	35.13 ± 0.60^d^	41.58 ± 0.66^d^	38.95 ± 0.49^c^	90.33 ± 0.40^b^	91.22 ± 0.13^c^	8.27 ± 1.64^a^	7.9 ± 1.35^b^	1.71 ± 0.03^a^	1.70 ± 0.04^b^
(b) DSW amendment										
Control	27.86 ± 0.36^a^	30.98 ± 0.15^a^	31.95 ± 0.35^a^	31.41 ± 0.51^a^	88.72 ± 0.78^a^	88.46 ± 0.25^a^	8.21 ± 0.63^a^	8.13 ± 1.45^a^	1.75 ± 0.05^c^	1.73 ± 0.03^c^
1 %	30.90 ± 0.32^b^	32.95 ± 1.27^b^	37.07 ± 0.37^c^	35.18 ± 0.42^b^	88.33 ± 0.35^a^	89.73 ± 0.45^b^	8.27 ± 1.78^a^	8.05 ± 1.74^a^	1.72 ± 0.08^b^	1.71 ± 0.07^b^
5 %	32.72 ± 0.45^c^	34.82 ± 0.28^c^	34.65 ± 0.36^b^	40.66 ± 0.44^d^	91.66 ± 0.50^c^	89.93 ± 0.45^b^	8.20 ± 0.94^a^	8.24 ± 1.63^a^	1.69 ± 0.05^a^	1.69 ± 0.08^a^
10 %	34.23 ± 0.60^d^	36.40 ± 0.36^d^	39.60 ± 0.34^d^	38.84 ± 4.78^c^	90.53 ± 0.40^b^	91.27 ± 0.46^c^	7.97 ± 1.23^a^	8.18 ± 1.77^a^	1.70 ± 0.04^a^	1.69 ± 0.02^a^

Value reported in the table are the average values for the fruiting bodies obtained from the three flushes

Data followed by the same *superscript* in each column are statistically not different from each other (*P* < 0.05) using DMRT

The total soluble sugar varied within the limits of 31.41–41.58 %, fat between 1.68 and 1.75 %, moisture content between 88.33 and 91.85 % and ash between narrow limits of 7.8 and 8.2 %. These values fairly coincide with the ones reported by Bisaria et al*.* (1987) and Ragunathan and Swaminathan (2003). However, no correlation could be drawn between the yield and the percentage content of protein, total soluble sugars and fat in the mushroom fruit bodies.

### Degradation of cellulose, hemicellulose and lignin components of sugarcane bagasse as a result of mushroom cultivation

Cellulose, hemicellulose and lignin were utilised by the mushrooms to varying extents (Table 5). After 50 days of incubation with *Pleurotus* spp.*,* the amended bagasse substrate was altered significantly. The percent degradation of each component varied with the treatments.

**Table 5:** Chemical analysis of bagasse substrate amended with DWW and DSW; 50 days after inoculation

	Hemicellulose Reduction (%)		Cellulose Reduction (%)		Lignin Reduction (%)	
	*P. flabellatus*	*P. sajor*-*caju*	*P. flabellatus*	*P. sajor*-*caju*	*P. flabellatus*	*P. sajor*-*caju*
(a) DWW amendment						
Control	22.80 ± 0.75^a^	24.03 ± 0.61^a^	15 ± 0.36^a^	14.8 ± 0.55^a^	20.80 ± 1.3^a^	18.27 ± 0.96^a^
1 %	33.25 ± 0.76^b^	28.97 ± 1.35^b^	19.03 ± 0.41^b^	17.73 ± 0.8^ab^	25.60 ± 1.21^b^	23.45 ± 0.69^b^
5 %	36.37 ± 0.82^c^	31.16 ± 2.32^b^	20.43 ± 1.07^b^	19.11 ± 1.41^b^	27.33 ± 0.60^b^	25.69 ± 1.04^c^
10 %	37.13 ± 0.87^c^	40.80 ± 2.0^d^	22.66 ± 1.15^c^	23.14 ± 0.87^c^	29.30 ± 0.56^c^	27.8 ± 0.21^c^
(b) DSW amendment						
Control	22.80 ± 0.75^a^	24.03 ± 0.61^a^	15 ± 0.36^a^	14.8 ± 0.55^a^	20.80 ± 1.3^a^	18.27 ± 0.96^a^
1 %	28.01 ± 0.58^b^	33.46 ± 1.15^c^	20.10 ± 0.86^b^	22.94 ± 1.71^b^	23.71 ± 0.53^b^	28.92 ± 0.43^b^
5 %	34.27 ± 0.76^d^	32.67 ± 0.95^c^	24 ± 0.81^c^	20.97 ± 1.33^b^	28.92 ± 0.45^c^	27.66 ± 2.21^b^
10 %	30.56 ± 0.96^c^	29.30 ± 0.92^b^	21.10 ± 0.87^b^	17 ± 0.70^a^	22.23 ± 1.05^ab^	22 ± 0.21^a^

Data followed by the same *superscript* in each column are statistically not different from each other (*P* < 0.05) using DMRT

According to Pant et al. (2006), dry-matter loss starts from the very first day of inoculation, whereas lignin degradation starts at a later stage. The fungus utilises the soluble carbohydrates in the initial stages, as a result of which, significant percentage dry-matter reduction occurs. After primary growth of fungus, lignin degradation starts and cellulose is freed from lignocellulosic complex and the digestibility increases continuously. Thus, soluble carbohydrate and hemicellulose are consumed as an energy source prior to cellulose and lignin, at the stage of mycelial growth (Carmen 2009). In our experiments also, both strains of *Pleurotus* spp. degraded hemicellulose and lignin more selectively than cellulose. Out of the weight losses of chemical components in substrate during culture, the decrease in hemicellulose was the most remarkable from spawn inoculation to harvest of the fruit-bodies (0–50 days) being highest (40.80 %) in *P. sajor*-*caju* grown on bagasse with 10 % DWW and lowest (22.80 %) in *P. flabellatus* control. Lignin degradation was highest (29.30 %) in *P. flabellatus* grown on bagasse amended with 10 % DWW and lowest (18.27 %) in *P. sajor*-*caju* control. Cellulose was the component which was degraded the least during the growth of mushrooms. These results can also be corroborated by the work of Mukherjee and Nandi (2004), Pant et al*.* (2006), Carmen (2009) and Isikhuemhen and Mikiashvili (2009). Various authors have also tried to establish a correlation between lignocellulosic degradation and biological efficiency (Isikhuemhen and Mikiashvili 2009; Isikhuemhen et al*.*^2008^; Wang et al. 2001). In our study, a significant correlation was obtained between the extent of degradation of these components (mainly hemicelluloses and lignin), substrate dry-matter loss and the biological efficiency within a particular species at different concentrations of an amendment used. Greater losses in the hemicellulose and lignin content lead to greater substrate dry-matter loss and hence improved yields/biological efficiency. These findings can again be well supported by the work of Pant et al. (2006).

## Conclusions

From the present study, we find that DWW and DSW effluents could be suitably used at low concentrations for enhancing the *Pleurotus* mushroom productivity. While *P. sajor*-*caju* (PS 1610) performed better on bagasse amended with DWW, *P. flabellatus* (PF 1832) was more suited to grow on DSW amended bagasse. Although these effluents could be used at very low concentrations (5 and 10 % for DSW and DWW, respectively) in the substrate combinations, mushroom cultivation still proved to be one of the highly simple, beneficial and an economic method for disposing off the agricultural residues, such as bagasse, along with effective utilisation of the industrial effluents which are generated in abundance annually. Other methods for bioremediation of the industrial effluents could be further explored.

For a particular mushroom species, the degradation of hemicellulose and lignin was in accordance with the substrate dry-matter loss and so were the yield and biological efficiency. Greater losses in the dry-matter lead to higher yields, and hence, improved biological efficiency. In addition, the mushroom fruit bodies obtained with the use of effluent amendments had a higher protein content compared to that of the controls. The study is important from the point of view of resource recovery. Thus, on the basis of higher yields, improved biological efficiency and richer protein content, DWW and DSW effluents (when used at lower concentrations) appear as a suitable option for substrate amendment of bagasse for *Pleurotus* species cultivation.
